# Super-Resolution Procedure for Target Responses in KOMPSAT-5 Images

**DOI:** 10.3390/s22197189

**Published:** 2022-09-22

**Authors:** Seung-Jae Lee, Sun-Gu Lee

**Affiliations:** Korea Aerospace Research Institute, 169-84, Gwahak-ro, Daejeon 34133, Korea

**Keywords:** KOMPSAT-5, SAR remote sensing, satellite SAR, super-resolution, target response

## Abstract

Recently, target analysis using satellite SAR images has received much attention in the area of satellite SAR remote sensing. Because the spatial resolution of the target response in the satellite SAR image is a main factor that has a large effect on target analysis performances, the improvement of the spatial resolution of target response is required to enhance the target analysis capability. However, the spatial resolution is already determined in the satellite SAR system design process. To solve the above problem, the super-resolution techniques that have been applied to radar images can be utilized. However, the application of the super-resolution techniques to the target response in the satellite SAR image is not simple due to the following reasons. First, the target’s motion induces severe blurring of the target response, which impedes the successful improvement of spatial resolution. Next, the zero-region in the frequency spectrum of the target image containing the target response also hinders the generation of the super-resolved image. To successfully improve spatial resolution of the satellite SAR image, the super-resolution techniques should be combined with proper preprocessing steps that can cope with the above two issues. In this paper, the whole super-resolution procedure for target responses in KOMPSAT-5 images is described. To the best of the authors’ knowledge, the description of the whole super-resolution procedure for target responses is the first ever attempt in the area of satellite SAR. First, a target image containing the target response is extracted from a large-scale KOMPSAT-5 image. Subsequently, the target image is transformed to be appropriate for the utilization of super-resolution techniques by proper preprocessing steps, considering the direction of super resolution and the motion of the target. Then, some super-resolution techniques are utilized to improve the spatial resolutions and qualities of the target images. The super-resolution performances of the proposed scheme are validated using various target images for point static, extended static, and extended moving targets. The novelties of this paper can be summarized as follows: (1) the practical design of whole super-resolution processing for real satellite SAR images; (2) the performance evaluation of super-resolution techniques on real satellite SAR images. The results show that the proposed scheme can led to noticeable improvements of spatial resolution of the target images for various types of targets with reliable computation times. In addition, the proposed scheme also enhanced PSLR, ISLR, and IC, leading to clearer scattering information of the principal scatterers. Consequently, the proposed method can assist in extracting more precise and meaningful information for targets represented in KOMPSAT-5 images, which means great potential for target recognition.

## 1. Introduction

Recently, in the area of satellite synthetic aperture radar (SAR) remote sensing, many studies have focused on target analysis, such as detection and classification of target responses [1,2,3,4,5,6,7,8]. Generally, the spatial resolution of a satellite SAR system has a large effect on the interpretation of the scattering mechanisms of the target in satellite SAR images [9]; therefore, it is a key factor in target analysis using satellite SAR images. The spatial resolution of satellite SAR images is directly associated with the 3 dB bandwidth of the impulse response functions (IRFs) of scatterers in satellite SAR images. Thus, as the spatial resolution improves in the range and azimuth directions, sharper IRFs can be obtained and the interferences among the IRFs are reduced, leading to more accurate scattering information (i.e., geometric locations and radar cross sections of principal scatterers) in satellite SAR images. Thus, the improvement of the spatial resolution of satellite SAR images plays a crucial role in improving target analysis capability using satellite SAR images. However, the spatial resolution of satellite SAR images has already been determined in the satellite SAR system design process, considering the operational objectives and application area of the corresponding satellite SAR mission. Specifically, the frequency bandwidth and synthetic aperture length are set to determine the range and azimuth resolutions, respectively; in general, they cannot be controlled by the user.

In the areas of airborne SAR and inverse SAR (ISAR), the super-resolution (SR) concept has been introduced in many studies [9,10,11,12,13,14,15] to generate super-resolved radar images for specific targets. The SR algorithms in [9,10,11,12,13,14,15] are based on high-resolution spectral estimation (SE) techniques, such as autoregressive (AR) model-based linear prediction (LP), multiple signal classification (MUSIC), estimation of signal parameters via rotational invariance techniques (ESPRIT), and relaxation (RELAX), which successfully generate super-resolved radar images for various targets, solving the limitations of predetermined spatial resolutions. In addition, some studies have demonstrated that super-resolved target images can enhance target recognition capabilities [16,17].

In [18], compressive sensing (CS), an up-to-date technology, was used to generate super-resolved radar images of targets. According to the CS theory [19,20], a certain signal can be effectively recovered from incomplete measurements whose sampling rates are lower than the Nyquist sampling rate if the signal is sparsely representable in redundant basis functions. Because the principal scattering centers of targets are sparsely distributed in a small part of the radar image domain, CS algorithms have been successfully used to recover target information from incomplete scattered field signals in the areas of SAR and ISAR imaging [21,22,23,24]. The CS concept can also be applied to generate super-resolved target images. If we assume that the received radar signals are incomplete measurements, the super-resolved target image can be generated by applying CS algorithms to the received radar signals [18].

The aforementioned SR techniques can be applied to satellite SAR images. In [25], the SR procedure was roughly described and applied to KOrea the SATellite-5 (KOMPSAT-5) image, which is a high-resolution satellite SAR image acquired in the *X*-band. Specifically, the Burg algorithm was applied to the target image containing the target response of a point target to improve the spatial resolution in the slant-range and azimuth directions. Among the many SE techniques, the Burg algorithm has been adopted because of its efficiency with respect to accuracy and complexity (computation time). The experiments in [25] demonstrated some improvements in the IRFs of the point target.

However, the experiments in [25] considered only a point target and a specific algorithm (i.e., the Burg algorithm), leading to limited performance assessments. Thus, further investigations are needed to analyze the applicability of SR techniques to KOMPSAT-5 images. In addition, the entire SR procedure must be refined for practical application. For example, the SR procedure for a moving target requires additional preprocessing steps, such as refocusing [26,27]. In addition, the zero-region removal in the frequency spectrum should be executed differently depending on the SR direction.

Therefore, we designed the entire SR procedure for target responses in KOMPSAT-5 images. To best of the authors’ knowledge, the description of whole SR processing for target responses is the first ever attempt in the area of satellite SAR. Briefly, target images containing different types of targets were first extracted from large-scale KOMPSAT-5 images. Next, appropriate preprocessing steps were applied to the target images based on their characteristics. Thereafter, the AR-model-based LP and CS algorithms were applied to the preprocessed target images to generate super-resolved target images. Then, in the experiments, the SR capabilities of the proposed scheme were evaluated using various target images for static, extended static, and extended moving targets in a number of different ways.

## 2. Generation of Super-Resolved Target Image from Large-Scale KOMPSAT-5 Image

### 2.1. Overall Flow of the Proposed Scheme

**Figure 1:**
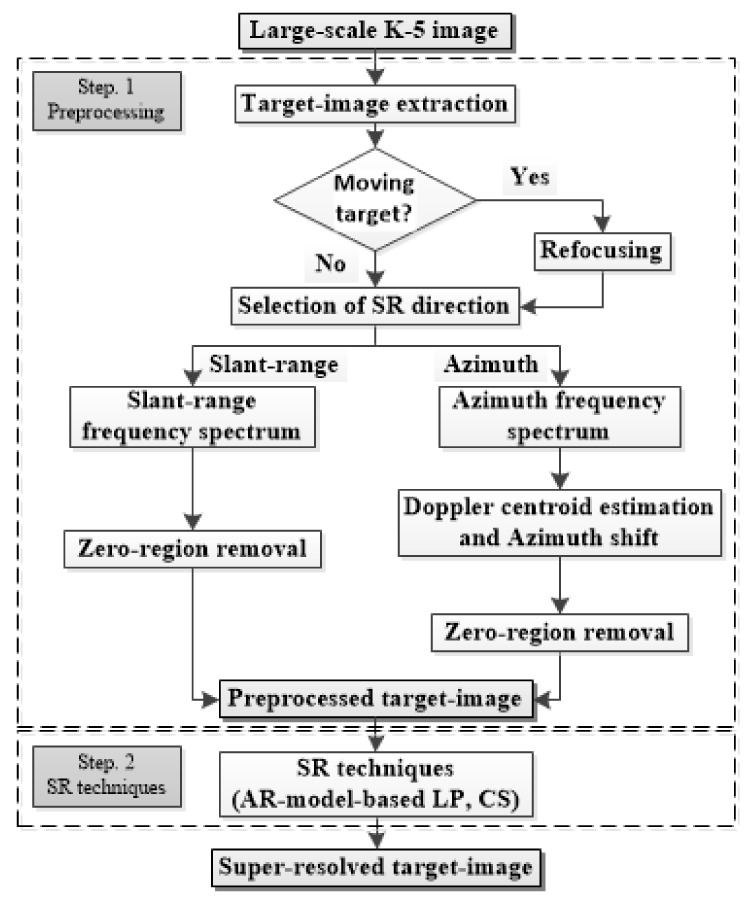
Overall flow of the proposed scheme.

The overall flow of the proposed scheme is illustrated in Figure 1. The proposed scheme consists of two steps: (1) preprocessing, and (2) utilization of SR techniques. In (1), the target image extracted from the KOMPSAT-5 image is transformed to be appropriate for the utilization of SR techniques. Then, the SR techniques are used to generate super-resolved target images in (2). In the next sections, the above-mentioned steps will be specifically explained.

### 2.2. SAR Signal Model of Target Image for the Proposed Scheme

According to the high-frequency scattering theory, a backscattered field in the high-frequency region can be represented as a sum of fields from a discrete set of independent scattering centers (SCs) on a target [28]. For simplicity, we utilize an undamped exponential model without the angle dependence as well as the frequency dependence term included in the geometrical theory of diffraction (GTD) model. Thus, the scattered field signals from I SCs at different look angles ϕ of target image can be modeled as [22]:

(1) $$s_{\phi ,f}=\sum_{i=1}^{I}a_{i}\exp(-j2k\sin\phi \cdot y_{i})\exp(-j2k\cos\phi \cdot x_{i})$$

where $a_{i}$ denotes the amplitude of the ith SC at $\left(x_{i},y_{i}\right)$ and k=2πf/c denotes the corresponding wavenumber at the frequency f. Let $f_{x}=f\cos\phi$ and $f_{y}=f\sin\phi$. Then, Equation (1) can be expressed as:

(2) $$s_{n_{az},n_{sl}}=\sum_{i=1}^{I}\exp⟮-j2\pi n_{az}\cdot \frac{y_{i}}{R_{y}}⟯\exp⟮-j2\pi n_{sl}\cdot \frac{x_{i}}{R_{x}}⟯$$

where $R_{y}=c/(2\Delta f_{y})$ and $R_{x}=c/(2\Delta f_{x})$ denote the maximum unambiguous range in the azimuth and slant-range directions. $n_{az}$ and $n_{sl}$ are indices to azimuth and slant range. If the two-dimensional (2D) SAR image domain is discretized by a 2D R×U grid, Equation (2) can be expressed as:

(3) $$s_{n_{az},n_{sl}}=\sum_{r=0}^{R-1}\sum_{u=0}^{U-1}a_{rq}\exp⟮-j\frac{2\pi }{R}n_{az}r⟯\exp⟮-j\frac{2\pi }{U}n_{sl}q⟯$$

When we consider only one direction (slant-range or azimuth direction) for the SR, the signal model in Equation (3) can be simplified as follows [24]:

(4) $$s_{n}=\sum_{i=0}^{I-1}a_{i}\exp⟮-j\frac{2\pi }{I}ni⟯$$

where *n* is $n_{az}$ or $n_{sl}$.

### 2.3. Preprocessing

To conduct SR processing, a small target image was first extracted from a large-scale KOMPSAT-5 image. Figure 1 shows examples of target images for static and moving targets extracted from KOMPSAT-5 images. The automatic identification system (AIS) information was used to verify the ship’s velocity.

**Figure 2:**
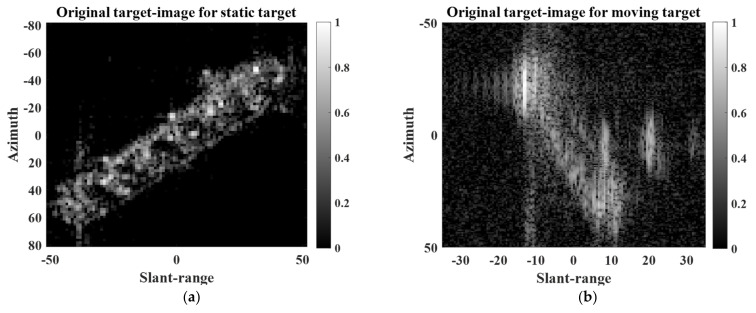
Examples of small target images: (**a**) static target, (**b**) moving target.

As shown in Figure 2a, if a target has no motion, then the target image contains a clear target response. In contrast, if a target has dynamic motion, the target response in the target image may be severely blurred owing to motion-induced phase errors in the scattered field signals, as shown in Figure 2b. In [26,27], the refocusing concept was introduced to remove phase errors and effectively mitigate the blurring effect of target responses. Thus, in this study, the refocusing strategy was applied to a target image if it contained the target response of a moving target with a blurring effect. Specifically, phase errors were removed based on the minimization of the Shannon entropy of the target image [29].

**Figure 3:**
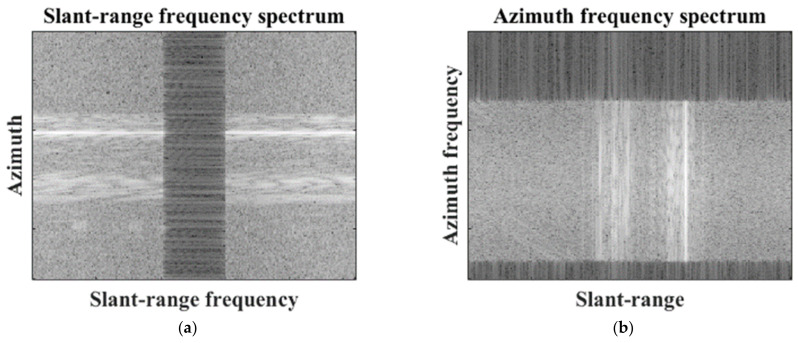
Frequency spectrum of target image. (**a**) Slant-range frequency spectrum, (**b**) azimuth frequency spectrum.

Next, the small target image is decompressed using a fast Fourier transform (FFT) along the SR direction in which the spatial resolution is improved, yielding the frequency spectra shown in Figure 3. In Figure 3, the frequency spectra contain zero-regions in the slant-range or azimuth directions, which are induced by oversampling in the SAR processor (SARP) [30].

To achieve high-quality SR results, the frequency spectrum must contain only continuous target information. However, the zero-regions break the continuity of the target information in the frequency spectrum, hindering the successful generation of super-resolved images. Thus, zero-regions should be removed in the SR direction. In the case of the slant-range frequency spectrum, the zero-regions are always located in the middle part of the spectrum by the characteristics of SAR processing, which can be directly removed using meta-information provided by SARP. Meanwhile, in the case of the azimuth frequency spectrum, the location of the zero-region is circularly shifted, depending on the Doppler centroid. Thus, the Doppler centroid is estimated to circularly shift the frequency spectrum in the azimuth direction such that the zero-regions are located in the middle part of the azimuth frequency spectrum. The zero-regions are then removed from the slant-range or azimuth frequency spectrum using the meta-information provided by SARP. In this study, we refer to the frequency spectrum whose zero-regions are removed as $S\in {\mathbb{C}}^{N_{az}\times N_{sl}}$, $N_{az}$, and $N_{sl}$, which denote the number of pixels in the azimuth (or azimuth frequency) and slant-range (or slant-range frequency) directions, respectively.

S was then used to generate the SR image using the following SR techniques.

### 2.4. SR Technique Using AR-Model-Based LP Algorithm

In the case of radar images, the spatial resolutions are inversely proportional to the frequency bandwidths in the slant-range and azimuth directions. The AR-model-based LP extends the frequency bandwidths of the scattered field signals using extrapolation and then generates a new target image with improved spatial resolution. 

Let the scattered field signal at a specific slant-range bin (in the case of the azimuth frequency spectrum) or azimuth bin (in the case of the slant-range frequency spectrum) be noted by $s_{n}$; n=1, 2, …, N, where N is either $N_{az}$ or $N_{sl}$. The AR model assumes that $s_{n}$ is a sum of undamped exponentials [11,16,17]. In the AR model, $s_{n}$ should satisfy the following forward and backward linear prediction conditions along the slant-range frequency or azimuth frequency directions:

(5) $${\hat{s}}_{n}=\left\{\begin{array}{c} -\sum_{i=1}^{k}a_{i}s_{n-i},\;\;\;\;\;n=k+1,\;k+2,\;\ldots ,\;N\\ -\sum_{i=1}^{k}a_{i}^{*}s_{n+i},\;\;\;\;\;n=1,\;2,\;\ldots ,\;N-k\;\;\;\;\;\;\; \end{array}\right.$$

where ∗ denotes the complex conjugate, N can be either $N_{sl}$ (in the case of the AR model in the slant-range frequency direction) or $N_{az}$ (in the case of the AR model in the azimuth frequency direction), $a_{i}$ denotes the coefficients of the AR model, k is the AR model order, and ${\hat{s}}_{n}$ is the estimated data using forward or backward prediction. The forward prediction error $e_{n}^{f}$ and backward prediction error $e_{n}^{b}$ can then be defined as follows:

(6) $$e_{n}^{f}={\left|s_{n}-{\hat{s}}_{n}\right|}^{2}={\left|\sum_{i=0}^{k}a_{i}s_{n-i}\right|}^{2},\;\;\;n=k+1,\;k+2,\;\ldots ,\;N,$$

(7) $$e_{n}^{b}={\left|s_{n}-{\hat{s}}_{n}\right|}^{2}={\left|\sum_{i=0}^{k}a_{i}^{*}s_{n+i}\right|}^{2},\;\;\;n=1,\;2,\;\ldots ,\;N-k$$

where $a_{0}=1$. The LP method determines the coefficients of the AR model $a_{i}$ to minimize the sum of the forward and backward prediction errors in Equations (6) and (7). Among the many LP methods, we adopted the Burg method [11] and modified covariance method (MCM) [11], which have been widely used in radar imaging [16,17]. In addition, we selected k=N/3 because it provides a robust estimation of $a_{i}$ [31]. 

**Figure 4:**
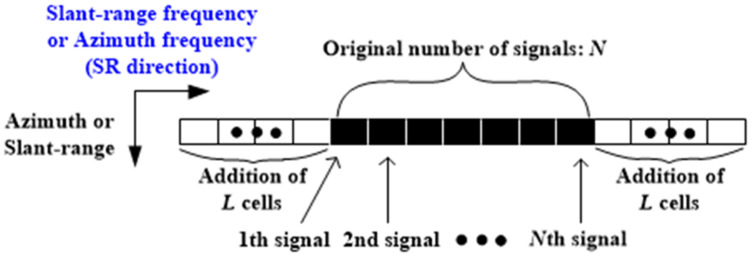
Increase in the number of cells in SR direction.

Once $a_{i}$ is obtained, the number of additional cells for extrapolation is determined as follows:

(8) $$L=\mathrm{Round}\left[0.5\times N\times \left(res_{be}/res_{af}-1\right)\right],$$

where $\mathrm{Round}\left[\cdot \right]$ denotes the round-off operator, $res_{be}$ and $res_{af}$ are the spatial resolutions in the slant-range or azimuth direction before and after the SR procedure, respectively. Note that LP assumes that L is proportional to the increment of the frequency bandwidth in the slant-range or azimuth direction after extrapolation [14]. Thus, L is crucial in determining the adjusted spatial resolution of the resulting super-resolved target image. Next, L cells were added to the first and last cells of $s_{n}$ along the SR direction, as shown in Figure 4. Thereafter, the scattered field signals of the 2L cells were estimated using $a_{i}$. The above steps are iterated for all slant-range or azimuth bins. Finally, inverse FFT (IFFT) is applied in the SR direction, yielding a super-resolved target image. In the case of the super-resolution procedure using Burg algorithm, the computational cost consists of one FFT, Burg algorithm, and one IFFT. The computational complexity of the FFT and the IFFT are O(NlogN) and O[(N+2L)log(N+2L)], respectively [32]. In addition, the Burg algorithm requires $3Nk-k^{2}-2N-k$ complex additions, $3Nk-k^{2}-N+3k$ complex multiplications, and k real divisions [33].

### 2.5. SR Technique Using CS Algorithm

In the area of radar imaging in the high-frequency region, a scattered field signal vector $\bar{z}\in {\mathbb{C}}^{P\times 1}$ can be represented as a linear combination of I columns in a Fourier dictionary $F\in {\mathbb{C}}^{P\times P}$ as follows:

(9) $$\bar{z}=F\bar{a}=\sum_{i=1}^{I}{\bar{f}}_{i}a(i)$$

where $\bar{a}\in {\mathbb{C}}^{P\times 1}$ denotes the scattering coefficient vector and ${\bar{f}}_{i}\in {\mathbb{C}}^{P\times 1}$ denotes the ith column of the matrix F. Because the scattering information of the target is concentrated in a small part of the radar image domain, c is generally small. When $\bar{a}$ has I non-zero elements, it is said to be I-*sparse*. If $\bar{z}$ is fully and uniformly sampled, with complete data, the unique solution of $\bar{a}$ can be easily estimated from Equation (9). However, in some cases, the radar system experiences missing data, leading to nonuniform undersampled scattered field signal vector $\bar{y}\in {\mathbb{C}}^{Q\times 1}$ (Q<P). In this case, the linear equation in Equation (9) is represented differently:

(10) $$\bar{y}=\Phi F\bar{a}=W\bar{a}=\sum_{i=1}^{I}{\bar{w}}_{i}a(i),$$

where $\Phi \in {\mathbb{C}}^{Q\times P}$ denotes the partial sensing matrix and $W\in {\mathbb{C}}^{Q\times P}$ denotes the partial Fourier dictionary. Because Q<P Equation (10) is an underdetermined system, leading to an infinite number of solutions for $\bar{a}$. With regard to digital signal processing, the sampling rates in Equation (10) do not satisfy the Nyquist sampling theory; thus, intact recovery of $\bar{a}$ from $\bar{y}$ is impossible with severe aliasing.

However, according to the CS theory, $\bar{a}$ can be successfully recovered from the limited measurements $\bar{y}$ if W satisfies the restricted isometry property (RIP) condition [19]. Because the partial Fourier dictionary W satisfies the RIP condition, the solution can be obtained by minimizing the $l_{0}$-norm of $\bar{a}$:

(11) $$(P_{0}):\;\underset{\bar{a}}{\min}{\left\|\bar{a}\right\|}_{0}\;\;\;\;\;\mathrm{subject}\;\mathrm{to}\;\;\;\;\;\bar{y}=W\bar{a},$$

where ${\left\|\cdot \right\|}_{0}$ refers to the $l_{0}$ norm of a vector (the number of non-zero elements). However, the optimization of nonconvex $\left(P_{0}\right)$ is an NP-hard problem that is extremely complex and difficult to solve [19,21,22]. To cope with this problem, many studies have utilized convex relaxation, which replaces the $l_{0}$ norm with the $l_{1}$ norm, as follows [19,20]:

(12) $$(P_{1}):\;\underset{\bar{a}}{\min}{\left\|\bar{a}\right\|}_{1}\;\;\;\;\;\mathrm{subject}\;\mathrm{to}\;\;\;\;\;\bar{y}=W\bar{a},$$

where ${\left\|\cdot \right\|}_{1}$ is the $l_{1}$ norm. In addition, the equality constraint $\bar{y}=W\bar{a}$ in Equation (12) is frequently replaced with a quadratic constraint to mitigate strictness, as follows [19,20]:

(13) $$(P_{1}^{\varepsilon }):\;\underset{\bar{a}}{\min}{\left\|a\right\|}_{1}\;\;\;\;\;\mathrm{subject}\;\mathrm{to}\;\;\;\;\;{\left\|\bar{y}-W\bar{a}\right\|}_{2}\leq \varepsilon ,$$

where ${\left\|\cdot \right\|}_{2}$ denotes the $l_{2}$ norm, and ε denotes the error tolerance, which is a small positive value.

In this study, we utilize Equations (12) and (13), which are widely known as basis pursuit (BP) and BP denoising (BPDN) [19,20,34], to generate super-resolved images. There are several reliable software packages that implement CS algorithms. In this study, we used $l_{1}$-magic software by Candes and Romberg [34]. In [34], Equation (12) can be recast as linear programs (LPs). Then, the LPs are solved using a generic path-following primal–dual method. In addition, Equation (13) can be recast as second-order cone programs (SOCPs). Then, the SOCPs are solved with a generic log-barrier algorithm. In Equation (13), ε is the parameter that affects both the computation time (CT) and accuracy of optimization. If ε is set to low, the CT is increased and the accuracy can become better, and vice versa. Thus, it is preferable to find ε to make the CT as small as possible while maximizing the accuracy. Thus, we tried to search the optimal ε heuristically, and then ε was set to 0.05 in this study. In addition, log-barrier tolerance, γ, which determines the number of log-barrier iterations, is set to 0.001, referring to [34]. Let the scattered field signal vector at a specific slant-range bin (in the case of the azimuth spectrum) or azimuth bin (in the case of the slant-range spectrum) in S be denoted by $\bar{s}\in {\mathbb{C}}^{N\times 1}$, where N is either $N_{sl}$ or $N_{az}$. We first consider $\bar{s}\in {\mathbb{C}}^{N\times 1}$ as an incomplete signal vector with undersampling [that is, $\bar{y}$ in Equation (10)], which consists of only part of the total scattered field signal vector $\bar{t}\in {\mathbb{C}}^{(N+2L)\times 1}$ [that is, $\bar{z}$ in Equation (9)]; L can be set by the user. Then, the 1D super-resolved scattering coefficient vector can be obtained by solving Equations (12) and (13) for $\bar{s}$ instead of $\bar{y}$. Finally, the above steps are iterated for all slant-range or azimuth bins, yielding a super-resolved target image. In the case of the super-resolution procedure using compressive sensing algorithm, the computational cost consists of one FFT [i.e., O(NlogN)], and compressive sensing algorithm. The computational complexity of the compressive sensing algorithm is influenced by several factors, such as problem sizes, parameter settings, and signal complexity [20].

## 3. Experimental Results

To evaluate the SR capacity of the proposed scheme, we conducted SR experiments using four target images for three different types of targets: point static, extended static, and extended moving.

### 3.1. SR Results for Point Static Target

**Figure 5:**
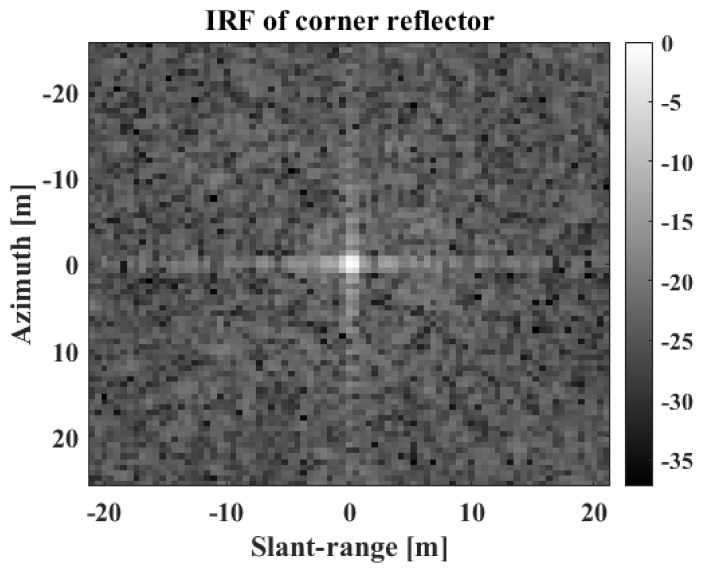
IRF of corner reflector.

To obtain the target image for a point target, KOMPSAT-5 observed a real corner reflector (CR) located at the KOMPSAT calibration site in Mongolia using spotlight mode and HH polarization. The target image was then extracted to contain only the impulse response function (IRF) of the CR, as shown in Figure 5.

A preprocessed target image was first generated to investigate the SR capability of the proposed scheme, and the preprocessed (PR) target image was first generated. Then, the spatial resolution of the preprocessed target image was intentionally worsened by reducing the slant-range or azimuth frequency bandwidth, leading to a low-resolution (LR) target image, as described in [25]. Let the ratio of the adjusted SR to the original SR be denoted as r. In this section, r is set to 1.6. Next, the AR-model-based LP and CS algorithms were applied to the LR target image in both the slant-range and azimuth directions. The slant-range cut and azimuth cut were then obtained by cutting the super-resolved target image at the center pixels in the slant-range and azimuth directions, respectively. Then, the 3 dB bandwidth, peak side-lobe ratio (PSLR), and integrated side-lobe ratio (ISLR) [35,36], which are widely used as the quality parameters of SAR images, were computed to quantify the SR capacity. The 3 dB bandwidth is the distance between the points with intensities 3 dB below the maximum intensity of the main lobe peak [35]. In addition, the PSLR is defined as the ratio of the peak amplitude of the most prominent side lobe to the peak amplitude of the main lobe, as in the following [35]:

(14) $$\mathrm{PSLR}=20\log_{10}\frac{Peak_{P-side}}{Peak_{main}},$$

where $Peak_{P-side}$ denotes the peak amplitude of the most prominent side lobe, and $Peak_{main}$ is the peak amplitude of the main lobe. In addition, the ISLR is the ratio of the total power in all the side lobes to the power in the main lobe, as in the following [35]:

(15) $$\mathrm{ISLR}=20\log_{10}\frac{Power_{A-side}}{Power_{main}}$$

where $Power_{A-side}$ denotes total power in all the side lobes, and $Power_{main}$ is the power in the main lobe.

**Figure 6:**
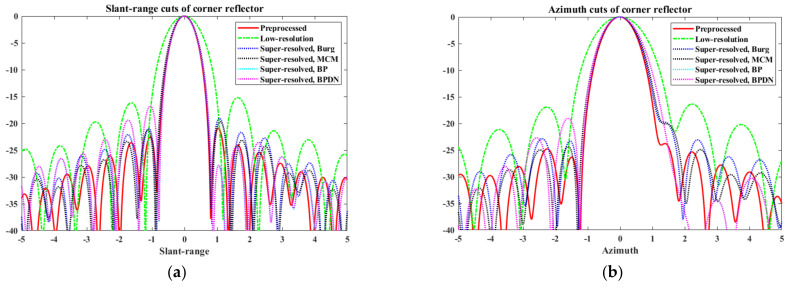
Slant-range cuts and azimuth cuts of PR, LR, and super-resolved target images: (**a**) slant-range cuts, (**b**) azimuth cuts.

Figure 6 shows the slant-range and azimuth cuts of the PR, LR, and super-resolved images.

In Figure 6, the solid red line denotes the slant-range and azimuth cuts of the PR target image; the dashed green line denotes the slant-range and azimuth cuts of the LR target image generated from the PR target image; the remaining four dotted lines denote the SR results. From Figure 6, the proposed scheme exhibits remarkable SR performance along both the slant range and azimuth directions. In particular, the main lobes of the super-resolved slant range and azimuth cuts approximately match those of the PR slant range and azimuth cuts. Quantitative comparisons of the quality parameters are summarized in Table 1 and Table 2. The results in Table 1 and Table 2 are obtained from the average values of 100 independent realizations to provide reliable performance evaluations for the BP and BPDN algorithms. In these tables, the super-resolved slant-range and azimuth cuts show significant improvements in the three quality parameters compared with the LR slant-range and azimuth cuts. In particular, as expected from Figure 6, all algorithms almost perfectly retrieved the 3 dB bandwidth of the PR target image from the LR target image. Thus, the proposed scheme successfully achieved the objective of SR (i.e., improvement of 3 dB bandwidth) for the KOMPSAT-5 image. In addition, the proposed scheme enhances the PSLR and ISLR of the LR target image. Although they cannot attain PSLR and ISLR equivalent to the PR target image, the qualities of the super-resolved target images are superior to those of the LR target images.

**Figure 7:**
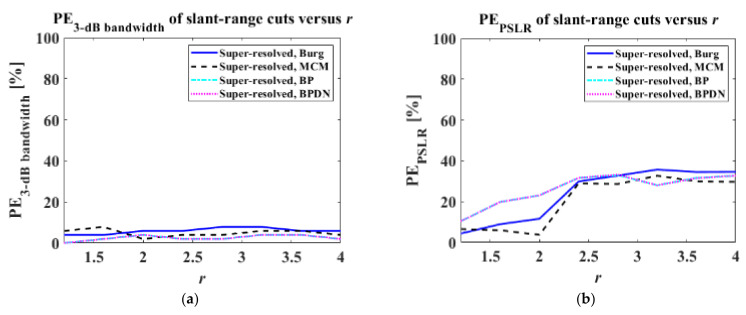

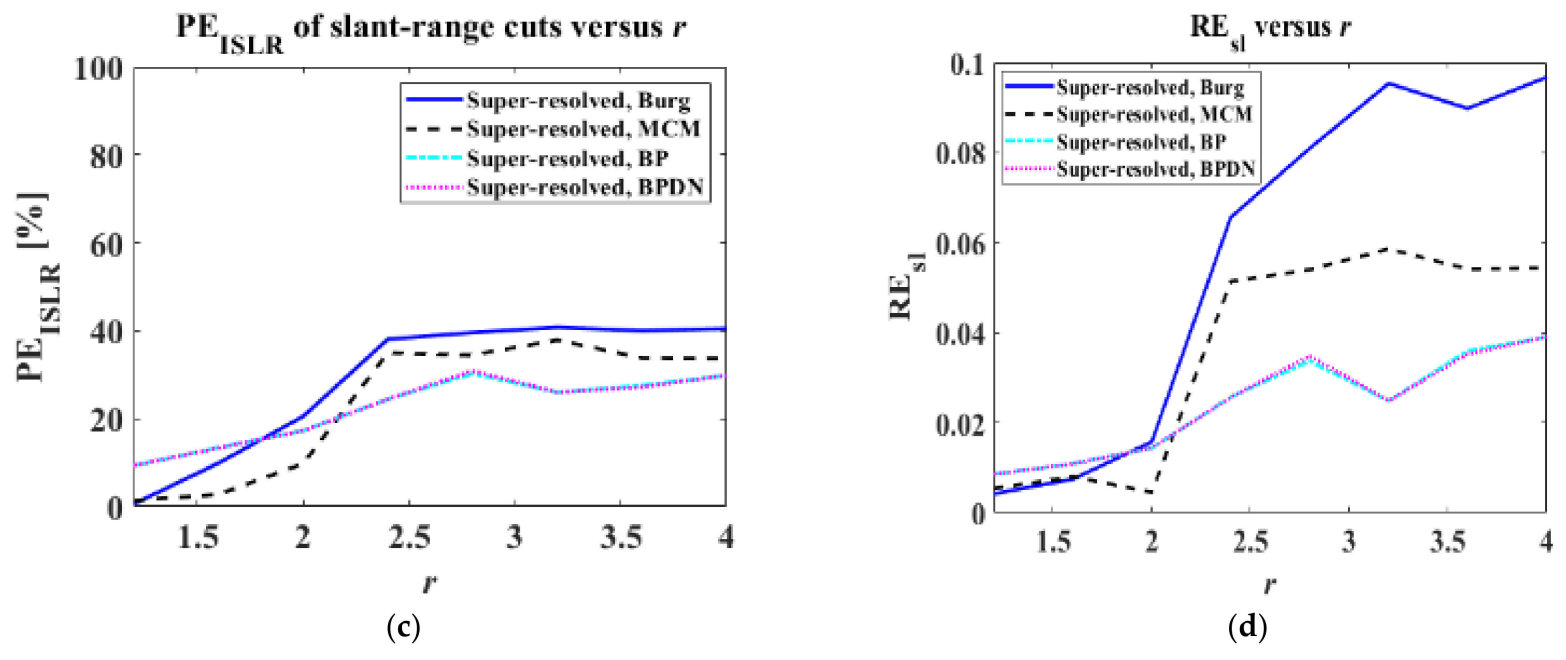
$PE_{3-dB\;bandwidth}$, $PE_{PSLR}$, $PE_{ISLR}$, and $RE_{sl}$ versus r: (**a**) $PE_{3-dB\;bandwidth}$, (**b**) $PE_{PSLR}$, (**c**) $PE_{ISLR}$, (**d**) $RE_{sl}$.

**Figure 8:**
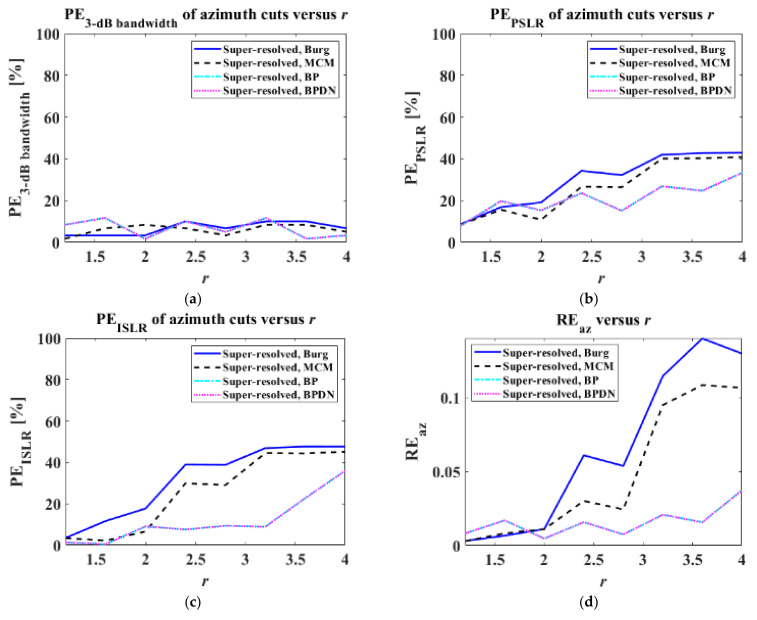
$PE_{3-dB\;bandwidth}$, $PE_{PSLR}$, $PE_{ISLR}$, $RE_{az}$ versus r for azimuth cuts: (**a**) $PE_{3-dB\;bandwidth}$, (**b**) $PE_{PSLR}$, (**c**) $PE_{ISLR}$, (**d**) $RE_{az}$.

The results in Table 1 and Table 2 demonstrate that the proposed scheme can effectively enhance the quality of the target image (i.e., the spatial resolution, PSLR, and ISLR). However, note that r is an important factor affecting SR capability. The capabilities of the four SR algorithms are sensitive to variations in r. To examine the SR performances of the four algorithms in detail, we define the relative error rate of the three quality parameters as follows:

(16) $$PE_{3-dB\;bandwidth,\;PSLR,\;ISLR}=\frac{\left|J^{PR}-J^{SR}\right|}{J^{PR}}\times 100,$$

where $J^{PR}$ denotes three quality parameters of the PR target image, and $J^{SR}$ denotes those of the super-resolved target images obtained using the four SR algorithms. In addition, the 1D relative errors of the slant range and azimuth cuts are defined as follows:

(17) $$RE_{sl,\;az}=\frac{\sum {\left(\left|I_{1D}^{PR}\right|-\left|I_{1D}^{SR}\right|\right)}^{2}}{\sum {\left(\left|I_{1D}^{PR}\right|\right)}^{2}},$$

where $I_{1D}^{PR}$ denotes the slant-range or azimuth cuts of the PR target image, $I_{1D}^{SR}$ denotes those of the super-resolved target images, and $\sum \left(\cdot \right)$ denotes the summation of all elements in a vector. $PE_{3-dB\;bandwidth,\;PSLR,\;ISLR}$ and $RE_{sl,\;az}$ in Equations (16) and (17) were then computed by varying r from 1.2 to 4 in increments of 0.4, as shown in Figure 7 and Figure 8.

In Figure 7, it can be observed that the $PE_{3-dB\;bandwidth}s$ of all algorithms is low over the entire range of r. This indicates that the main lobe of the slant-range cut of the PR target image can be successfully reconstructed from the LR slant-range cut, regardless of the variation in r. In particular, the BP and BPDN provide reliable $PE_{3-dB\;bandwidth}s$, the maximum of which is just lower than 4%. In the case of $PE_{PSLR}$, $PE_{ISLR}$, and $RE_{sl}$, SR performance worsens as r increases. For $PE_{PSLR}$, the AR-model-based LPs yield better performances than the CS techniques when r<2.4, and their performances become similar when r>2.4. In the case of $PE_{ISLR}$ and $RE_{sl}$, the Burg and MCM algorithms also show better performance than the CS techniques when r<2. However, CS techniques lead to lower (better) $PE_{ISLR}s$ and $RE_{sl}s$ when r>2. In particular, it is remarkable that the $RE_{sl}s$ of the Burg and MCM algorithms rapidly increased during r>2.

In Figure 8, as is the case with slant-range cuts, all algorithms exhibit reasonable $PE_{3-dB\;bandwidth}s$. In addition, $PE_{PSLR}s$, $PE_{ISLR}s$, and $RE_{az}s$ tend to deteriorate as r increases. For $PE_{PSLR}$, $PE_{ISLR}$, and $RE_{az}$, the AR-model-based LPs exhibit worse results than the CS techniques for r>2. In particular, the $RE_{az}s$ of the Burg and MCM algorithms significantly increase when r>2.

In short, in the case of a point static target, all SR algorithms yielded reliable results during r<2. In addition, the CS techniques produced more robust SR results than the AR-model-based LPs during r>2.

### 3.2. SR Results for Extended Targets

To analyze the SR performance for extended targets, we used two target images (i.e., extended static target and extended moving target) extracted from two different large-scale KOMPSAT-5 images, as shown in Figure 2. The KOMPSAT-5 image in Figure 2a was obtained using stripmap mode and HH polarization, whereas the KOMPSAT-5 image in Figure 2b was obtained using the spotlight mode and HH polarization.

As in the case of the point target, after the two target images were preprocessed according to Figure 1, the spatial resolutions of the two PR target images were deliberately degraded using r=1.6. The proposed SR scheme was then applied to the LR target image in both the slant-range and azimuth directions.

**Figure 9:**
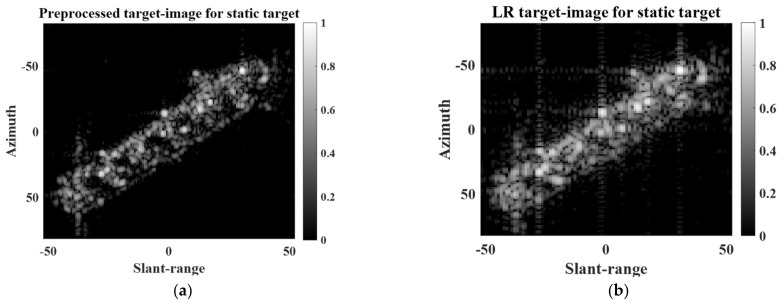

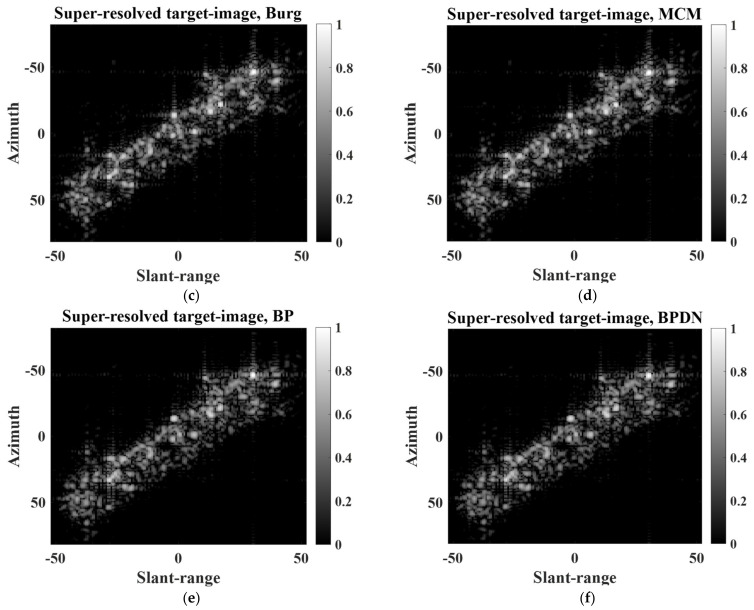
PR, LR, and super-resolved target images of extended static target: (**a**) PR, (**b**) LR, (**c**) Burg, (**d**) MCM, (**e**) BP, (**f**) BPDN.

Figure 9 shows the PR, LR, and super-resolved target images of the extended static target. As shown in Figure 9b, the quality of the LR target image is much lower than that of the PR target image. This is because (1) 3 dB bandwidths of IRFs corresponding to scattering centers deteriorate (widen) and (2) interference among IRFs increases [16]. Then, the target response of the ship is focused on improving the 3 dB bandwidths of the IRFs and reducing the interference among the IRFs, as shown in Figure 9c–f.

**Figure 10:**
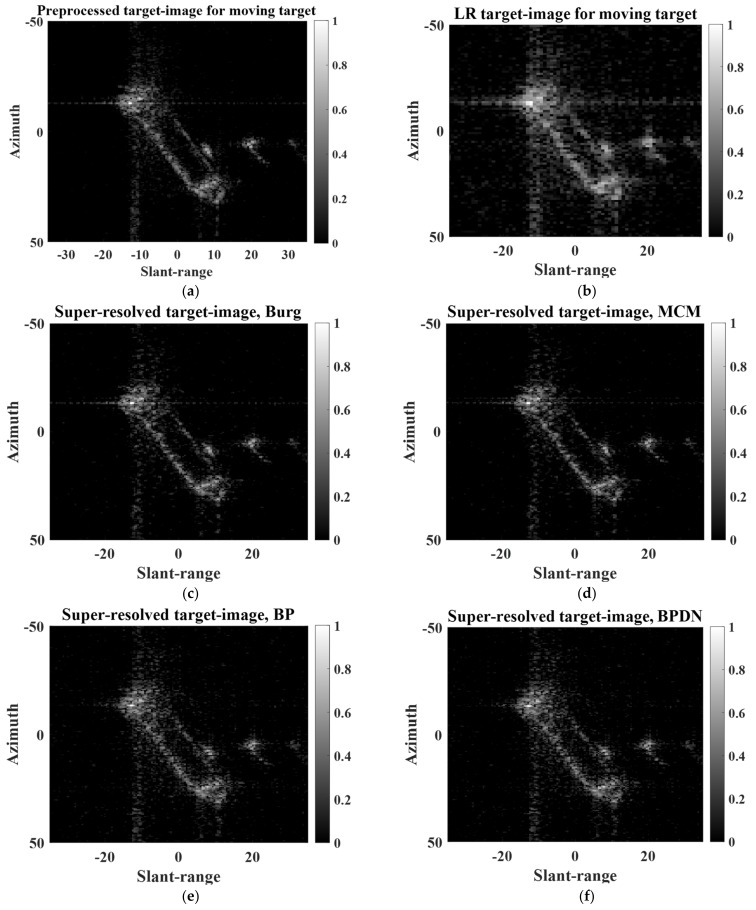
PR, LR, and super-resolved target images of extended moving target: (**a**) PR, (**b**) LR, (**c**) Burg, (**d**) MCM, (**e**) BP, (**f**) BPDN.

In addition, Figure 10 shows the PR, LR, and super-resolved target images of the extended moving target. Note that in the case of the moving target, the PR target image in Figure 10a differs considerably from the original target image in Figure 2b. This is because the refocusing method was applied to Figure 2b to achieve an intact response of the moving target. As shown in Figure 10, the proposed scheme successfully improves the quality of the LR target image. In particular, it appears that the qualities of Figure 10c,d are comparable to those of the PR target image.

Unlike in the case of the point target, the SR performance of the extended target cannot be quantitatively evaluated using SAR quality parameters. This is because the extended target consists of a large number of scattering centers, leading to arbitrary interference among the IRFs, which impedes the exact computation of the quality parameters.

Thus, as alternatives, we adopted Shannon entropy (SE) and image contrast (IC), which are widely used to evaluate the focus quality of SAR image [23,24,28]. The SE and IC can be written as follows [28]:

(18) $$\mathrm{SE}=\sum \sum \frac{{\left|I_{2D}\right|}^{2}}{S}\cdot \ln\frac{S}{{\left|I_{2D}\right|}^{2}},$$

(19) $$\mathrm{IC}=\frac{\sigma \left[{\left|I_{2D}\right|}^{2}\right]}{E\left[{\left|I_{2D}\right|}^{2}\right]},$$

where $I_{2D}$ denotes a 2D image, $\sum \sum \left(\cdot \right)$ denotes the summation of all the elements in a matrix, and $S=\sum {\sum \left|I_{2D}\right|}^{2}$. In addition, E[⋅] and σ[⋅] are the mean and standard deviation.

Generally, a lower SE and higher IC imply better focus quality of the SAR image. In addition, the improvement in focus quality can imply the improvement of 3 dB bandwidths of IRFs and a reduction in interference among IRFs, provided that other SAR imaging parameters are the same, as in the case of the PR and LR target images.

Table 3 and Table 4 show the SEs and ICs of the target images in Figure 9 and Figure 10, respectively. The results in Table 3 and Table 4 are obtained from the average values of 100 independent realizations to provide reliable performance evaluations for the BP and BPDN algorithms. As expected, the LR target images have much higher SEs and lower ICs than those of the PR target images. Meanwhile, the SEs and ICs of the super-resolved target images were effectively improved. For both case of static and moving targets, the Burg algorithm shows outstanding SEs and ICs, which are comparable to those of the PR target images.

In addition, the computation time (CT) for each super-resolved target image was measured to investigate the applicability of our scheme in real situations. For this, MATLAB programs and a PC with its CPU clock speed of 3.7 GHz were used (the MATLAB program is not optimized to obtain its best computation speed).

Table 5 and Table 6 show the CTs for super-resolved target images in Figure 9 and Figure 10. The results in Table 5 and Table 6 are obtained from the average values of 100 independent realizations to provide reliable performance evaluations for the BP and BPDN algorithms. As seen in Table 5 and Table 6, the proposed scheme has reliable CTs. In particular, the CT is just 0.05 s for the extended static target, when the Burg algorithm is chosen as the SR technique. Considering that our equipment and software are not optimized for data processing, the proposed scheme has large potential to be used for real systems.

Furthermore, the 2D relative error is defined to compare the SR performances of the four algorithms, as follows [22]:

(20) $$RE_{2D}=\frac{\sum \sum {\left(\left|I_{2D}^{PR}\right|-\left|I_{2D}^{SR}\right|\right)}^{2}}{\sum \sum {\left(\left|I_{2D}^{PR}\right|\right)}^{2}},$$

where $I_{2D}^{PR}$ denotes the PR target image, $I_{2D}^{SR}$ denotes the super-resolved target images, and $\sum \sum \left(\cdot \right)$ denotes the summation of all the elements in a matrix. Then, $RE_{2D}$ was computed for target images of extended targets, varying r from 1.2 to 4 in steps of 0.4.

**Figure 11:**
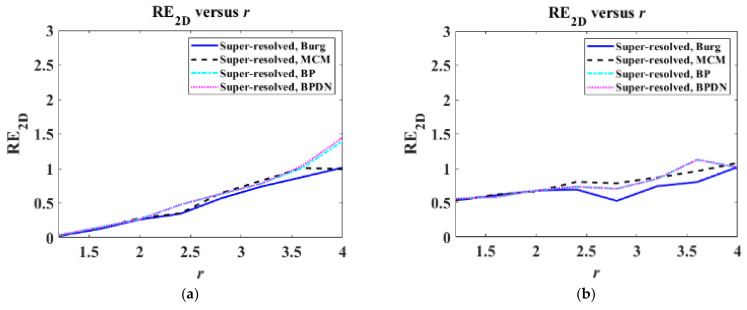
$RE_{2D}$ versus r for target images of extended targets: (**a**) static target and (**b**) moving target.

Figure 11 shows $RE_{2D}$ versus r for target images of extended targets. In Figure 11, all algorithms yield similar performances in the entire range of r, showing continuous growth of $RE_{2D}$. Among them, the Burg algorithms show the best performances versus r.

From Table 3 and Table 4 and Figure 11, we can observe that the SR performances of the Burg algorithm are better than those of the other algorithms in the case of the extended target used in this study.

**Figure 12:**
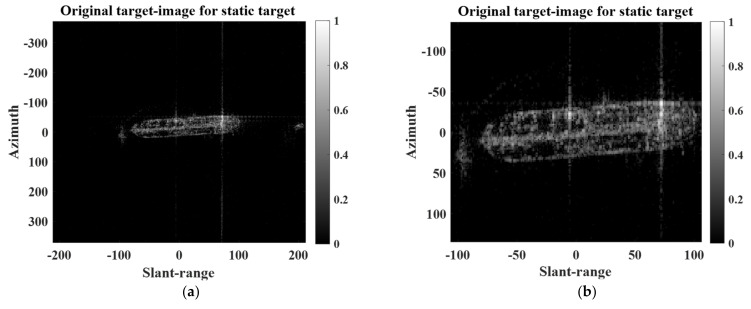
Target images of the extended static target having different χ: (**a**) χ=6.18 % and (**b**) χ=20.6 %.

**Figure 13:**
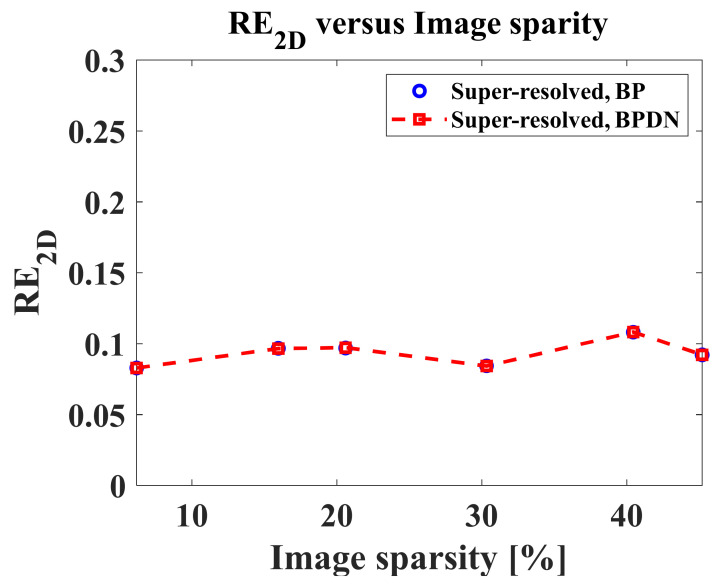
$RE_{2D}$ versus χ for target images of extended moving targets in Figure 12.

In our study, we adopted two super-resolution techniques, namely the AR-model-based LP algorithms and the compressive sensing algorithms. In the case of the AR-model-based LP algorithms, the sparsity of the target image does not affect the super-resolution performances theoretically. Meanwhile, in the case of compressive sensing algorithms, the sparsity can affect the super-resolution performances. Actually, most target images can be sparsely representable, because the target response is concentrated in a small part of the target image. However, the sparsity of the target images can be varied depending on target detection algorithms, which determine the region of interest containing the target response in different ways. Thus, we additionally investigated the super-resolution performances of the proposed scheme using CS algorithms in the case of different image sparsity. Let the ratio of the number of pixels corresponding to target response to the total number of pixels in the target image, χ, be the image sparsity. The pixels of the target responses are determined using the constant false alarm rate (CFAR) detector [1]. For experiments, we first extracted another target image of a specific extended static target from the large-scale KOMPSAT-5 image, as shown in Figure 12a. The χ of Figure 12a is 6.18%. Next, the χ of the target image was artificially adjusted by cropping the target image, as if the region of interest (ROI) for target response was changed. For example, Figure 12b is the resulting target image whose χ is 20.6%. Then, $RE_{2D}s$ for CS-based SR procedure versus χ were computed when r=1.6, as shown in Figure 13. In Figure 13, χs were 6.18, 15.96, 20.6, 30.33, 40.45, and 45.21%. As seen in Figure 13, the relative errors do not seriously change until the image sparsity reaches 45.21%. Consequently, it is expected that the proposed scheme can give stable performances in real situations that the image sparsity can be varied by the target detection algorithms.

## 4. Discussion

**Figure 14:**
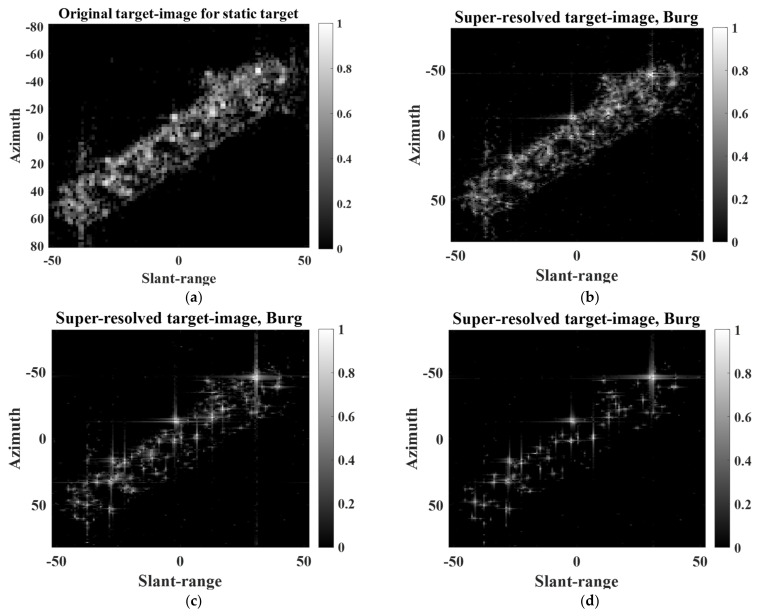
SR results for Figure 2a: (**a**) original, (**b**) α=3, (**c**) α=5, (**d**) α=7.

In Section 3, it is demonstrated that the proposed scheme is useful for improving the quality (i.e., 3 dB bandwidth, PSLR, ISLR, and IC) of target images extracted from a large-scale KOMPSAT-5 image. Although the four SR techniques result in some differences in their performances, they mostly work well for various types of targets. We think that the main application of the proposed scheme may be target recognition using satellite SAR images, because the super-resolved images can represent the scattering information of the main scatterers more clearly, as reported in [16,17]. Thus, our future work will use the proposed scheme for SAR target recognition. Let the ratio of the original SR to the improved SR be denoted as α. To investigate the potential of the proposed method for target detection and classification, we applied the proposed scheme to Figure 2a by varying α from 3 to 7 with steps of 2, as shown in Figure 14. Among the four algorithms, the Burg algorithm was chosen as the SR technique of the proposed scheme because it exhibited the best SR performance for extended targets in Section 3. In Figure 14, it can be observed that the proposed scheme substantially enhances the quality of the original target image. Consequently, the SR results provide more precise and delicate information about principal scatterers, effectively removing the messy parts of the target responses. Thus, we expect that the proposed method has great potential for target recognition.

We can consider utilizing the other spectral estimation techniques (e.g., subspace-based methods such as MUSIC [13] or ESPRIT [14]), which have been used in the area of radar signal processing (specifically, direction-of-arrival (DOA) estimation), for step 2 in the proposed scheme (i.e., super-resolution algorithm), instead of the AR-model-based LP algorithms. However, the subspace-based methods require much more computation times than AR-model-based LP algorithm [33,37]. In addition, their accuracies are very sensitive to the estimation of the number of sources, which is difficult for extended targets. Furthermore, if those are adopted as the super-resolution algorithm for step 2 in the proposed scheme, the characteristics of the impulse response functions (IRFs) in the resulting target images is completely lost; this may lead to critical degradation of SAR target recognition performances. Thus, we think it is desirable to use AR-model-based LP algorithms as the super-resolution algorithm to increase the efficiency for the main application of the proposed scheme such as near-real-time SAR target recognition.

## 5. Conclusions

In this study, we present a detailed SR procedure for target responses in KOMPSAT-5 images. In Section 3 and Section 4, the use of the proposed scheme led to remarkable improvements in the quality (i.e., 3 dB bandwidth, PSLR, ISLR, and IC) of the target images for various types of targets. Interestingly, the proposed scheme can enhance not only the spatial resolution (i.e., 3 dB bandwidth) but also PSLR, ISLR, and IC, leading to clearer scattering information of the principal scatterers. This implies that the proposed method can assist in extracting more precise and meaningful information for targets represented in KOMPSAT-5 images. Furthermore, the concept of the proposed scheme can be easily extended to other satellite SAR images, such as ICEEYE, Capella, COSMO-SkyMed, and KOMPSAT-6, if the preprocessing steps are slightly adjusted depending on the characteristics of those images. Thus, we expect that the proposed scheme will lead to improvements in target recognition capability using various satellite SAR images.

## Figures and Tables

**Table 1:** Comparison of three quality parameters of slant-range cuts.

	Measure		
	3 dB Bandwidth [m]	PSLR [dB]	ISLR [dB]
PR	0.67	−20.93	−16.73
LR	1.02	−15.21	−12.38
Burg	0.69	−19.06	−15.08
MCM	0.72	−19.69	−16.27
BP	0.68	−16.78	−14.48
BPDN	0.68	−16.79	−14.5

**Table 2:** Comparison of three quality parameters of azimuth cuts.

	Measure		
	3 dB Bandwidth [m]	PSLR [dB]	ISLR [dB]
PR	0.96	−23.76	−18.89
LR	1.44	−16.35	−13.15
Burg	0.99	−19.77	−16.68
MCM	1.02	−20.06	−18.48
BP	1.07	−19.07	−18.98
BPDN	1.07	−19.07	−18.98

**Table 3:** SEs and ICs of target images of extended static target.

	Target Image					
	PR	LR	Burg	MCM	BP	BPDN
SE	6.98	7.48	6.98	7.02	7.09	7.1
IC	9.92	7.03	9.3	9.1	9.3	9.22

**Table 4:** SEs and ICs of target images of extended moving target.

	Target Image					
	PR	LR	Burg	MCM	BP	BPDN
SE	5.06	5.69	4.96	5.07	5.41	5.41
IC	41.87	28.83	41.21	39.59	35.48	35.48

**Table 5:** CTs for super-resolved target images of extended static target.

	Target Image			
	Burg	MCM	BP	BPDN
CT (s)	0.05	0.06	0.18	0.29

**Table 6:** CTs for super-resolved target images of extended moving target.

	Target Image			
	Burg	MCM	BP	BPDN
CT (s)	0.12	0.17	1.01	1.39
